# Geographical distribution of Burkholderia pseudomallei in Taiwanese croplands and the influence of bacterial community interactions on its incubation viability

**DOI:** 10.1371/journal.pntd.0013640

**Published:** 2025-10-22

**Authors:** Kuang-Ying Chen, Kuang-Yueh Chen, Hsin-Ping Hu, Ching-Hao Teng, Hsi-Hsun Lin, Tzu-Hang Chen, Yao-Shen Chen, Bing-Mu Hsu, Hau-Ren Chen

**Affiliations:** 1 Department of Biomedical Sciences, National Chung Cheng University, Chiayi, Taiwan; 2 Department of Physics, National Kaohsiung Normal University, Kaohsiung, Taiwan; 3 Department of Biotechnology, National Kaohsiung Normal University, Kaohsiung, Taiwan; 4 Department of Molecular Medicine, National Cheng Kung University, Tainan, Taiwan; 5 Department of Infectious Diseases, E-Da Hospital/I-Shou University, Kaohsiung, Taiwan; 6 Department of Clinical Medicine, National Yang Ming Chiao Tung University, Taipei, Taiwan; 7 Department of Internal Medicine, Kaohsiung Veterans General Hospital, Kaohsiung, Taiwan; 8 Department of Medicine, National Sun Yat-sen University, Kaohsiung, Taiwan; 9 Department of Earth and Environmental Sciences, National Chung Cheng University, Chiayi, Taiwan; Colorado State University, UNITED STATES OF AMERICA

## Abstract

*Burkholderia pseudomallei* is a soil-borne pathogen responsible for melioidosis, a potentially fatal disease. While endemic melioidosis in Taiwan is marked by both clinical cases and environmental detection, a comprehensive environmental survey has been lacking. A nationwide cropland survey using *B. pseudomallei-*specific *orf2-*PCR revealed regional positivity rates of 2.1% in northern, 8.2% in central, 15.1% in southern, and 9.8% in eastern Taiwan, with the highest PCR positivity and cumulative melioidosis incidence (12.14 cases per 100,000 people from 2003 to 2024) observed in the south. Vertical profiling showed a higher DNA detection rate at a depth of 60 cm, with increased surface-level detection during rainy periods and a decline after sunny conditions, particularly in the southern area. Identical molecular sequence types across layers suggested upward movement from deeper soil. However, viable bacteria were not consistently recovered from PCR-positive samples, indicating that bacterial dynamics during incubation may influence *B. pseudomallei* viability. To investigate this, full-length 16S rDNA sequencing and community analysis revealed inverse abundance patterns between *B. pseudomallei* and *B. multivorans*, *B. cenocepacia*, and B. *vietnamiensis* during incubation. In vitro assays confirmed strong antagonism by *B. multivorans* filtered supernatants against the growth of *B. pseudomallei,* while *B. cenocepacia* and *B. vietnamiensis* had weaker or no effects. These findings reveal distinct regional and vertical patterns of *B. pseudomallei* DNA in croplands and highlight the potential influence of bacterial competition on its viability during incubation.

## Author summary

*Burkholderia pseudomallei* is a soil-dwelling bacterium that causes melioidosis, a potentially fatal infectious disease in humans and animals. Although melioidosis is endemic in Taiwan, nationwide environmental data on the distribution of *B. pseudomallei* have been limited. We conducted a large-scale cropland survey across Taiwan using a species-specific PCR method to detect *B. pseudomallei* DNA. The results revealed regional differences, with the highest detection rate and melioidosis incidence in southern Taiwan. DNA was more abundant in deeper soil layers (60 cm), but surface detection increased after rainfall, especially in the south. Notably, viable *B. pseudomallei* were not always recovered from PCR-positive samples, suggesting that other microbes may affect *B. pseudomallei* survival during enrichment. Bacterial community analysis identified an inverse relationship between *B. pseudomallei* and other *Burkholderia* species, particularly *B. multivorans*. Laboratory assays showed that *B. multivorans* strongly inhibited the growth of *B. pseudomalle*i through secreted compounds. These findings provide new insights into the environmental distribution of *B. pseudomallei* and suggest that bacterial interactions may influence its viability, with potential implications for disease risk assessment and environmental surveillance strategies.

## Introduction

*Burkholderia pseudomallei* is a Gram-negative saprophytic bacterium commonly isolated from soil and water in tropical and subtropical endemic regions. It is the etiologic agent of melioidosis, a potentially fatal disease that is often underdiagnosed or overlooked in areas with limited diagnostic capabilities or low public awareness [1–2]. Human melioidosis is primarily acquired through the subcutaneous contact, inhalation, or ingestion of *B. pseudomallei*, while human-to-human or animal-to-human transmission is rare [3]. The severity of melioidosis is influenced by factors such as exposure to large inocula of *B. pseudomallei*, infection by highly virulent strains, and host comorbidities such as diabetes and chronic kidney disease [1,4].

In endemic regions such as Ubon Ratchathani in northeastern Thailand, Darwin in the Top End of the Northern Territory in Australia, and Kaohsiung in southern Taiwan, *B. pseudomallei* has been consistently detected from soil, which serves as a natural reservoir for the bacterium [5–7]. Climatic changes, in particular, have been shown to be associated with the emergence of melioidosis [8–9]. Correlations between the presence of *B. pseudomallei* in the environment, the incidence of melioidosis in humans and animals, and seropositive rates in local populations have been documented [10–12]. To date, the classification of melioidosis-endemic regions has been primarily based on the environmental isolation of *B. pseudomallei* and the occurrence of non–travel–associated clinical cases in tropical and subtropical areas [13–14].

The high environmental isolation rates of *B. pseudomallei* highlight the need for increased clinical awareness of melioidosis in regions where the disease may occur. Nonetheless, systematic investigations into the environmental distribution of *B. pseudomallei* remain incomplete in many regions, including parts of Taiwan [7]. Furthermore, the vertical distribution of *B. pseudomallei* in soils has been reported down to 30–90 cm, and even as deep as at 100–200 cm below the surface, challenging the likelihood of frequent human exposure [7,15,16]. Comparing isolation rates across heterogeneous soil, geographic locations, and time periods reveals that detecting *B. pseudomallei* at the surface remains challenging, even with established consensus protocols for environmental sampling [17–18].

To enable the rapid analysis of large numbers of soil samples, PCR-based techniques were developed and applied for the specific detection and quantification of *B. pseudomallei* in environmental surveillance [7,19,20]. Challenges remain, as soils with high PCR-positive rates did not consistently yield *B. pseudomallei* isolates [7,20], suggesting that unidentified factors may influence changes in the bacterial population during cultivation. Addressing this issue could help bridge the gap between PCR detection and successful bacterial recovery.

From 2003 to 2024, a total of 790 melioidosis cases have been reported in Taiwan, with 82.6% of cases occurring in southern Taiwan, which is recognized as an endemic area due to higher incidence rates and the presence of *B. pseudomallei* in the environment [21]. In contrast, only five cases of melioidosis have been reported in eastern Taiwan, a region with limited healthcare infrastructure and no prior investigations into the environmental distribution of *B. pseudomallei*. This study aims to explore the geographical distribution of *B. pseudomallei* reservoirs in cropped soils across Taiwan, assess the vertical distribution of the bacteria in PCR-positive locations during a rainy event, and identify potential biological factors that may explain the failure to isolate the bacterium from PCR-positive soil samples during environmental surveys.

## Materials and methods

### Ethics statement

All procedures were approved by the Institutional Biosafety Committee of Kaohsiung Veterans General Hospital (VGH-1081226–1); and National Kaohsiung Normal University (NKNU-18/23–001).

### Microorganisms

*B. vietnamiensis* NKNU04, *B. multivorans* NKNU07, *B. cenocepacia* NKNU05, and *B. pseudomallei* VGH27 used in this study were isolated from the environment and identified using biochemical and molecular methods (see below). Viable *B. pseudomallei* were handled in an airflow-controlled laboratory (BSL-3 level, at Kaohsiung Veterans General Hospital).

### Random sampling strategy

The random sampling aimed to identify the locations of *B. pseudomallei* soil reservoirs across Taiwan. Sampling was conducted using Geographic Information System (GIS) data along the public road network, maintaining a straight-line interval of 0.3 to 3 km. Sampling sites were selected in agricultural land, while urban areas, protected zones, undisturbed lands, and restricted-access areas were excluded. Sampling took place from October 2022 to March 2023, a period of relatively stable precipitation in Taiwan. Considering that the 0–45 cm soil layer is disturbed by tillage practices, sampling was conducted to a depth of 60 cm in rice fields, herbaceous plant areas (e.g., vegetables and commercial flowers), and fallow land (typically covered with grass) to ensure the soil remained undisturbed and had not been recently exposed to sunlight. In fruit tree orchards, sampling was conducted to a depth of 30 cm, as tillage is not required.

At each sampling site, three individual holes were dug 1–5 m apart and 500 g of soil was collected from each hole. The digger (10 cm in diameter) was disinfected with 70% alcohol between collections to prevent cross-contamination. Soil samples were placed in sterile bags and promptly transported to the laboratory, where DNA extraction was carried out in the following days. Sites positive for *B. pseudomallei* DNA were defined by the presence of a positive PCR result in at least one of the three holes (see DNA extraction and PCR methods below).

### Fixed-interval and vertical sampling

**Fig 1:**
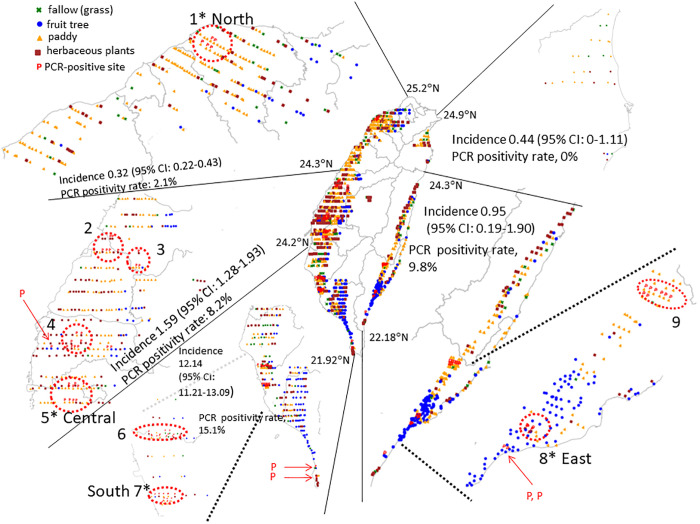
Sampling Sites, Soil PCR Positivity, and Cumulative Incidence of Melioidosis in Taiwan. The sampling sites were selected using a random strategy along the public road network. Symbols indicating plantation types, sampling sites and PCR-positive results are shown in the upper-left corner. The cumulative incidence of melioidosis (2003–2024), and the PCR positivity rate (October 2022–March 2023) are presented. Asterisks (*) above a number indicates sites selected for vertical distribution analysis in different regions. The basemap was adapted from open-access shapefiles provided by the Taiwan Government Open Data Platform (https://data.gov.tw, Open Government Data License, version 1.0).

Based on the PCR-positive results from the random sampling strategy described above, fields were selected from the northern (Cluster 1), central (Cluster 5), southern (Cluster 7), and eastern (Cluster 8) regions of Taiwan (see Fig 1 for cluster positions). To facilitate sampling, surface vegetation was gently cleared, and the ground was lightly leveled to ensure consistent soil access across sites while minimizing disturbance to subsurface layers. Sampling sites were determined using GIS and arranged in fixed-interval grids of 0.64 m^2^ within a 64 m² field, resulting in a total of 100 sampling squares per field. Sampling was conducted four times: following an initial sunny day, a rainy day, and then the first and second subsequent sunny days (which may not have been consecutive). A sunny day was defined as having no rainfall, a UV index >7, and a sunshine rate >35%, while a rainy day was defined as having rainfall >40 mm, a UV index <7, and a sunshine rate <35% in this study. All sampling was conducted from July to September 2023. Climatic information was obtained in real time from a climate station near the sampling field (https://codis.cwa.gov.tw/StationData?target=station; stations 467050, 467480, 467441, and 467660).

Soil samples (10 g for isolation; 2 g for DNA extraction) were collected from the land surface (0–10 cm, as sun-exposed soil), as well as from depths of 30 cm (disturbed soil) and 60 cm (undisturbed soil) below the surface. Additionally, backup soil was collected at each sampling site and stored at room temperature (70% humidity) for future bacterial community structure analysis. Isolation of *B. pseudomallei* and total DNA extraction were performed immediately after soil collection (see Isolation and PCR methods below). Culture-negative for *B. pseudomallei* was defined as the failure to yield the bacterium in three independent isolation attempts.

### Bacterial Community Structure Analysis Sampling

Following the results of a fixed-interval survey of the southern land surface (Cluster 7) on a rainy day, bacterial community structure analysis was conducted based on the following findings: a PCR-positive soil sample that did not yield *B. pseudomallei* at (6,4), a PCR-positive site with culture confirmation at (6,5), and a PCR- and culture-negative site at (7,4), according to x-y coordinates (see S1 Fig for coordinates). To minimize the impact of prior sampling, each new sampling point was located 10 cm from the center of the previous hole, and 500 g of soil was collected per site. Before bacterial community analysis, the PCR and culture results of the soil samples were reconfirmed.

### DNA Extraction from environmental samples

Soil samples (2 g or 10 g), collected through random or fixed-interval strategies or for bacterial community structure analysis, were suspended in sterile water at a 1:1 ratio and placed on a rotator (120 rpm) at 42°C overnight. The following day, 1 mL (from 2 g samples) or 5 mL (from 10 g samples) of homogenized mixture was inoculated into 10-fold volume of Ashdown’s broth (10 g/L Trypticase soy broth, 4% (v/v) glycerol, 5 mg/L crystal violet, 50 mg/L neutral red, and 8 mg/L gentamicin) and incubated for 2 h (for all environmental surveys) or 36 h (only for bacterial community structure analysis).

Soil pellets from Ashdown’s broth cultures were obtained by centrifugation at 16,000 × g for 10 min at room temperature using an Eppendorf centrifuge (Eppendorf, Hamburg, Germany). DNA was then extracted from the pellets using the DNeasy PowerSoil Kit (Qiagen, Hilden, Germany) according to the manufacturer’s protocol. Briefly, 0.25 g of soil pellets was subjected to chemical and mechanical lysis in a zirconium bead tube containing lysis buffer. Cell disruption was performed by vortexing using a bead tube adapter. Inhibitory substances were removed using proprietary commercial reagents, and nucleic acids were bound to a silica membrane using a chaotropic binding buffer. After a two-step wash, genomic DNA was eluted in 10 mM Tris buffer and stored at –20°C.

### Cultures, PCR, qPCR, and Biochemical Identification

Approximately 10 g of soil was suspended in 10 mL of sterile water and placed on a rotator (120 rpm) at 42°C overnight. The following day, 5 mL of homogenized mixture was inoculated into 50 mL of Ashdown’s broth at 42°C for 7 d. Streaking cultures or plating counts with serial dilution (n = 3, each culture) on Ashdown’s medium were performed daily for up to 7 d. Each suspected colony was cultured in Luria-Bertani (LB) broth at 37°C for 18–24 h and then subjected to biochemical identification, DNA purification, and PCR amplification.

For molecular identification, the DNA was extracted from bacterial pellets obtained from LB broth cultures after growth from a single colony, using the DNeasy Blood & Tissue Kit (Qiagen), following the protocol for Gram-negative bacteria. Bacterial pellets, obtained by centrifugation at 16,000 × g for 10 min from overnight cultures, were subjected to enzymatic lysis, followed by proteinase K digestion and column-based purification. DNA was eluted in AE buffer (Qiagen) and stored at –20°C.

A *B. pseudomallei* PCR amplification targeting the 115-bp *orf2* region of the type III secretion system (TTS1) was performed using purified DNA, with 45 cycles of 15 sec at 95°C and 15 sec at 59°C [19]. For quantitative PCR (qPCR), only purified DNA was used. The assay employed specific primers (BpTT4176F: 5′-CGTCTCTATACTGTCGAGCAATCG-3′; BpTT4290R: 5′-CGTGCACACCGGTCAGTATC-3′) and a fluorogenic probe (5′-CCGGAATCTGGATCACCACCACTTTCC-3′) in a final reaction volume of 25 µL. qPCR assays were performed using a 7900HT Fast Real-Time PCR System (Applied Biosystems, Inc., Foster City, CA), following a previously described protocol [22].

Genomic DNA from *B. multivorans*, *B. cenocepacia*, and *B. vietnamiensis* was purified from suspected colonies grown in LB broth*.* Both full-length 16S rDNA (primer; F, 5’-AGAGTTTGATCMTGGCTCAG-3’; R, 5’-TACCTTGTTACGACTT-3’) and *recA* gene (primers; BCR1, 5’-TGACCGCCGAGAAGAGCA-3’; BCR2, 5’-CTCTTCTTCGTCCATCGCCTCA-3’) were amplified. The PCR conditions consisted of 35 cycles, with denaturation at 96°C for 1 min, annealing at 56°C for 1 min, and extension at 72°C for 1.5 min [23]. Sequence analysis revealed >99.9% similarity to known sequences, leading to the identification of the bacteria as *B. vietnamiensis* NKNU04, *B. cenocepacia* NKNU05, and *B. multivorans* NKNU07, which were used in this study.

Biochemical identification of all isolates was performed using biochemical test profiles (API 20NE; bioMérieux, Marcy l’Etoile, France).

In the pre-test, over 96% and 100% of soil samples (n = 30, per endemic area) imported from melioidosis-endemic areas—Darwin in the Top End of the Northern Territory, Australia, and Ubon Ratchathani in northeastern Thailand, respectively—yielded *B. pseudomallei* isolates following the isolation protocols used in this study (S1 Table).

### Bacterial community structure analysis

The bacterial community structure was analyzed using total DNA extracted after 2 and 36 h of incubation in Ashdown’s broth. A total of 36 samples were included for 16S rDNA sequencing, representing 6 individual soils across three soil categories: (1) PCR- and culture-positive, (2) PCR-positive but culture-negative*,* and (3) PCR- and culture-negative for *B. pseudomallei*, at both 2- and 36-h incubation time points. DNA quantification was performed with the Qubit dsDNA HS Assay Kit (ThermoFisher Scientific, San Jose, CA, USA), and quality was assessed with the Agilent 4200 TapeStation system (Agilent Technologies, Palo Alto, CA, USA), as part of services provided by Welgene Inc. (Taipei, Taiwan).

The 16S Long Read Kit (Loop Genomics, San Jose, CA, USA) was used to construct a full-length 16S rDNA amplicon library. Library sequencing was performed on an Illumina NovaSeq platform (Illumina, San Diego, CA, USA) in paired-end 150 bp mode. Raw short reads were processed on a cloud-based platform maintained by Loop Genomics, which assembled them into high-quality consensus contigs. Synthetic long-read (SLR) sequence data were analyzed using the Divisive Amplicon Denoising Algorithm (DADA2). Sequences were screened for the presence of forward and reverse primers and trimmed to appropriate full-length 16S rDNA sizes, ranging from 1400 bp to 1600 bp. Amplicon Sequence Variants (ASVs), representing exact and unique DNA sequences, were generated by DADA2 and used for analysis in this study. Heat Tree visualizations were created using an R-based program for each time point (2 and 36 h), with each tree representing a combined analysis of 6 individual samples per condition. Each sample was analyzed separately to generate ASV tables, and the merged output was used to construct the Heat Tree, allowing comparison of overall bacterial community composition across distinct conditions. Node colors represent sequence count abundance, and node sizes correspond to the number of distinct taxa.

Additionally, the top 14 populations (each comprising over 1% of the total community) were selected from the ASV tables to observe individual changes in total population at 2- and 36-h incubation across the following groups: PCR- and culture-positive, PCR-positive but culture-negative*,* and PCR- and culture-negative for *B. pseudomallei* (n = 6 per group × 2 time points).

### Antagonistic assay

The supernatants of *B. vietnamiensis* NKNU04, *B. cenocepacia* NKNU05, and *B. multivorans* NKNU07 were obtained by culturing each strain in 50 mL of LB broth at 37°C for 48 h, followed by filtration through 0.22 μm membrane filters (Costar Inc., Cambridge, MA, USA). The incubation temperature was chosen based on the optimal growth rates of all tested strains. *B. pseudomallei* VGH27 was inoculated into the filtered supernatants at a final concentration of 5.5 × 10^4^-1.0x10^5^ CFU/mL and incubated at 37°C for 96 h (n = 6, per group). As controls, *B. vietnamiensis* NKNU04, *B. cenocepacia* NKNU05, and *B. multivorans* NKNU07 were inoculated into their respective supernatants in parallel experiments. Bacterial numbers (CFU/mL) were determined at defined intervals using serial dilution and plating.

### Multilocus sequence typing (MLST)

The housekeeping genes (*ace, gltB, lepA, lipA, nark, ndh*, and *gmhD*) were amplified and sequenced using primers as previously reported [24]. The alleles at each locus were assigned by comparing the sequences with those available on the website (https://pubmlst.org/). The STs (sequence types) were determined using the allelic profile database. Phylogenetic analysis was performed using MEGA11 (Maximum Likelihood Tree method; open source: https://www.megasoftware.net/) and the resulting tree images were generated using iTOL (branch lengths ignored; open source: https://itol.embl.de/).

### Statistics

The descriptive statistic used for melioidosis in this study was cumulative incidence over the period 2003–2024. Spearman’s rank correlation was used to assess the relationship between PCR positivity and soil depth, as well as the inverse association between *B. pseudomallei* and other *Burkholderia* species during incubation. Correlations between PCR positivity at different soil layers and climatic factors—such as rainfall, sunshine rate, and ultraviolet index (UVI)—were evaluated using Poisson or negative binomial regression models, selected based on Akaike Information Criterion (AIC) values. Data from antagonistic assays were expressed as mean ± SD (standard deviation; n = 6 per group). Differences between groups were assessed using the Mann-Whitney U test. A *p*-value of <0.05 was considered statistically significant.

## Results

### Geographical distribution of *B. pseudomallei*

Geographical distribution in Taiwan was surveyed using a random sampling strategy. As a result, the PCR-positive signals specific to *B. pseudomallei* DNA were detected in 14 sampling areas. Of these, 9 areas exhibited spatial clustering of positive sites (northern: n = 1; central: n = 4; southern: n = 2; eastern: n = 2) while the remaining 5 sites were scattered and isolated, with no neighboring sites testing positive (central: n = 1; southern: n = 2; eastern: n = 2) (Fig 1). The sampling sizes, areas, plantation types, and average DNA concentration in each region are shown in S2 Table. Cumulative melioidosis incidence from 2003 to 2024 is shown in S3 Table. The high proportion of PCR-positive samples (15.1%) coincided with the high cumulative melioidosis incidence (12.14 cases per 100,000 people; 95% CI:11.21–13.09) in southern Taiwan, compared to the PCR positivity rates and incidence in northern (2.1% vs. 0.32 cases; 95% CI:0.22–0.43), central (8.2% vs. 1.59 cases; 95% CI:1.28–1.93), and eastern (9.8% vs. 0.95 cases; 95% CI:0.19–1.90) Taiwan. No PCR positivity was detected in the northeastern regions, where the incidence was 0.44 cases per 100,000 people (95% CI:0–1.11) (Fig 1).

### Vertical distribution of *B. pseudomallei*

**Fig 2:**
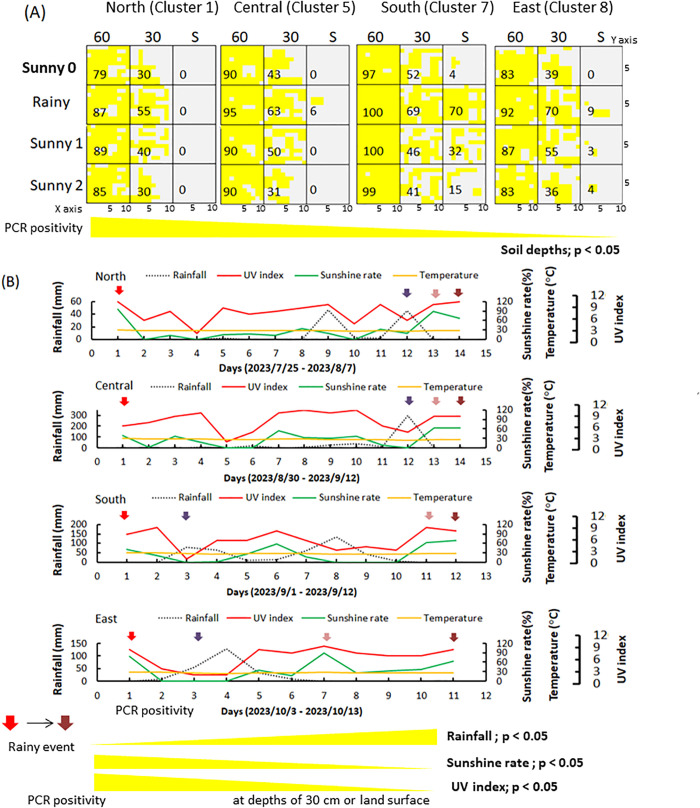
Vertical Distribution of *B. pseudomallei* and Climatic Conditions. A fixed-interval and vertical sampling strategy (0.64 m² per square in a 64 m² field) was used to detect *B. pseudomallei*-specific *orf2* amplicons at depths of 60 cm, 30 cm, and the land surface (S, 0–10 cm). Sampling was conducted in the northern (Cluster 1), central (Cluster 5), southern (Cluster 7), and eastern (Cluster 8) regions of Taiwan during a rainy event, which included an initial sunny day (Sunny 0), a rainy day (Rainy), and the first (Sunny 1) and second (Sunny 2) sunny days after rainfall. Yellow and grey indicate PCR-positive and PCR-negative sites, respectively. The percentages of PCR-positive sites per 100 squares are shown above and a simplified graphic illustrating the correlation between PCR positivity and depths (*p* < 0.05) is shown below (A). The corresponding climatic conditions (rainfall, temperature, UV index, and sunshine rates) at each sampling region during the study period (shown above), along with a simplified summary of PCR positivity in relation to individual climatic factors are presented below (*p* < 0.05). Colored arrows indicate the sampling time: red for Sunny 0, purple for Rainy, light brown for Sunny 1, and dark brown for Sunny 2 (B).

To investigate whether *B. pseudomallei*-specific DNA accumulates near the land surface during rainfall, and whether PCR-positive sites reflect the presence of viable bacteria, vertical soil sampling was conducted at four representative regions (Cluster 1, north; Cluster 5, central; Cluster 7, south; Cluster 8, east) across Taiwan at depths of 60 cm, 30 cm, and the land surface. Initially, PCR positivity was highest at 60 cm and declined with decreasing depth, with almost no detection at the land surface (Fig 2A; spatial distribution; PCR positivity vs. soil depth, *p* < 0.05). Climatic changes during the sampling period, including rainfall, sunshine, and UV exposure, are summarized in Fig 2B. During rainy events, PCR-positive detection increased significantly at shallower depths (30 cm: *p* < 0.01) and at the land surface (0–10 cm: *p* < 0.01). This increase was positively associated with rainfall but negatively associated with sunshine rate and UVI across all regions (Fig 2B). Surface-level detection was most pronounced in the south (Cluster 7), where it peaked during rainfall and declined in the days under sunny conditions (Fig 2A). Regional data are detailed in S2 (north), S3 (central), S4 (south), and S5 (east) Figs.

**Fig 3:**
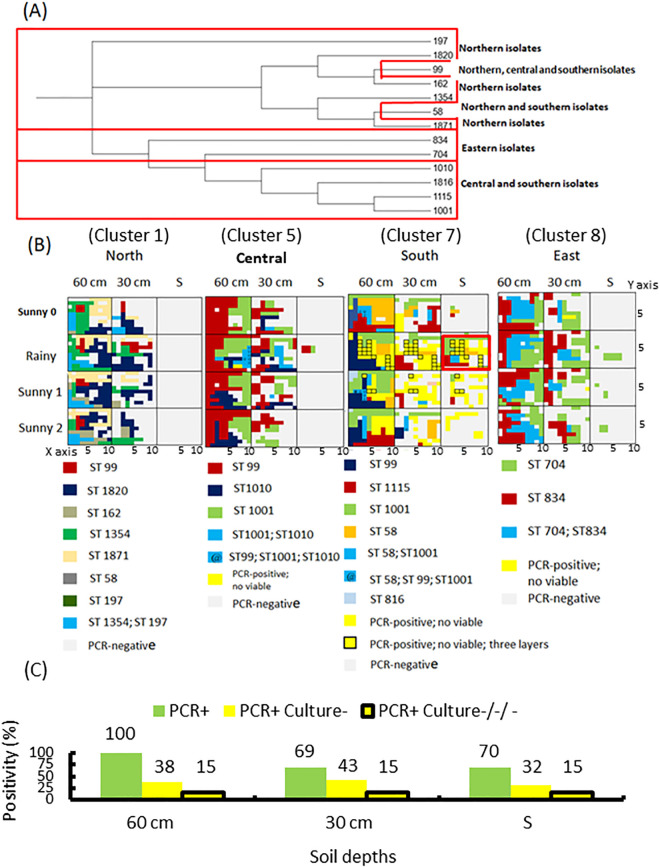
Distribution of Sequence Types (STs) of *B. pseudomallei* Across Sampling Regions and Soil Depths. The phylogenetic tree of *B. pseudomallei* sequencing types (STs) identified across Taiwan is shown (A). The vertical distribution of each ST (represented by color symbols at the bottom) is presented for depths of 60 cm, 30 cm, and surface layer (S, 0–10 cm) in the northern (Cluster 1), central (Cluster 5), southern (Cluster 7), and eastern (Cluster 8) regions. Sampling was conducted during a rainy event, including an initial sunny day (Sunny 0), a rainy day (Rainy), and the first (Sunny 1) and second (Sunny 2) sunny days following rainfall. Each square represents a 0.64 m² soil sampling unit. A red outline in the southern region highlights the area selected for the representative data shown below (B). Representative data from the red-marked area showing the proportions of PCR-positive samples (PCR + , green), PCR-positive but culture-negative samples in a single soil layer (PCR+ Culture − , yellow), and PCR-positive but culture-negative samples across all three soil layers (PCR+ Culture − / − / − , yellow with a solid line). The full distribution of PCR amplification across all soil layers and regions is provided in S2-S5 Figs.

Culture-based isolation confirmed the presence of *B. pseudomallei*, with sequence types (STs) showing geographical patterns: ST58, ST99, ST162, ST197, ST1354, ST1820, and ST1871 were found in the north; ST99, ST1001, ST1010, ST1115, and ST1816 in the central region; ST58, ST99, ST1001, ST1010, ST1115, and ST1816 in the south; and ST704 and ST834 in the east (Fig 3A). Several STs—including ST58, ST99, ST704, ST1001, and ST1115—were detected across all soil layers during rainy days (Fig 3B; spatial distribution of each ST across tested regions; numerical data provided in S2–S5 Figs), suggesting upward movement or redistribution during rainfall events.

Despite high PCR detection rates, viable *B. pseudomallei* could not be recovered from a substantial proportion of PCR-positive samples, particularly in southern soils during rainy periods. Approximately 32–43% of PCR-positive samples at various depths in the south failed to yield viable isolates, including 15% of samples that were PCR-positive but culture-negative across all three soil layers (Fig 3C). In contrast, PCR-positive but culture-negative samples were rare (<3%) and occurred sporadically in inconsistent soil layers in other studied regions (Regional data are detailed in S2-S5 Figs).

### Bacterial Community Structure

To assess whether the decline of *B. pseudomallei* during incubation in Ashdown’s broth coincided with broader shifts in the bacterial community—such as changes in diversity or the relative abundance of specific taxa—the community structure was analyzed using full-length 16S rDNA sequencing. Soil samples were grouped into three categories: (1) PCR- and culture-positive, (2) PCR-positive but culture-negative, and (3) PCR- and culture-negative for *B. pseudomallei*.

**Fig 4:**
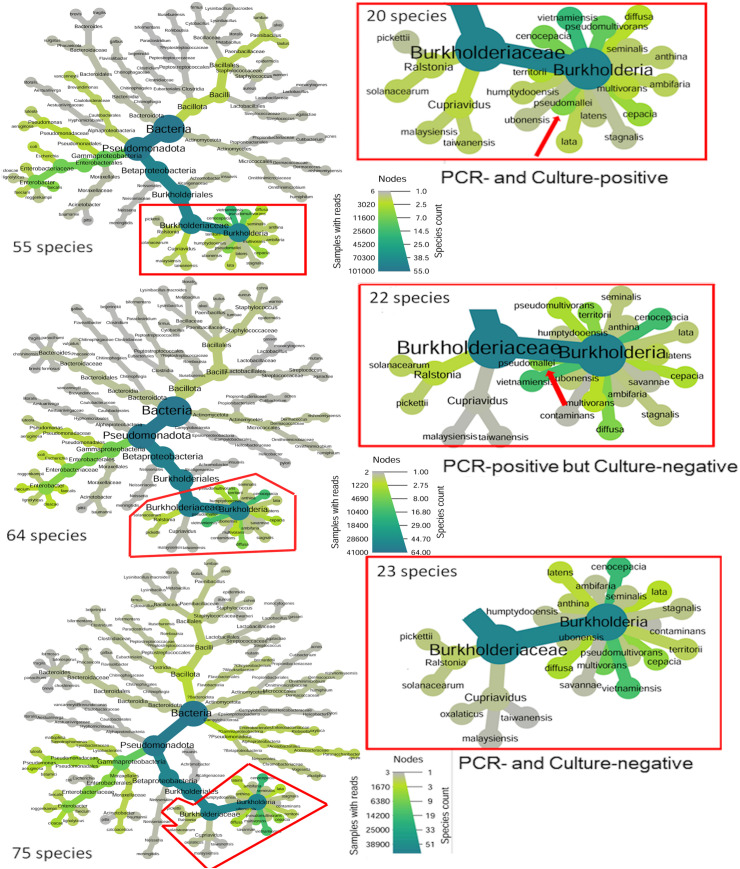
Bacterial Community Structures After 2 h of Incubation in Ashdown’s Broth. Soil supernatants prepared with sterile water and incubated overnight at 42°C were further cultured in Ashdown’s selective broth for 2 h. Total DNA was extracted and analyzed using full-length 16S rDNA sequencing. A Heat Tree visualization, generated using an R-based program from a combination of 6 individual datasets, depicts the bacterial community composition across 3 soil groups: (1) PCR- and culture-positive, (2) PCR-positive but culture-negative and (3) PCR- and culture-negative for *B. pseudomallei*. The *Burkholderiaceae* family is highlighted by a red frame on the right. Red arrows indicate the position of *B. pseudomallei.* Node color intensity reflects the relative abundance (read counts), and node size corresponds to the number of taxa.

**Fig 5:**
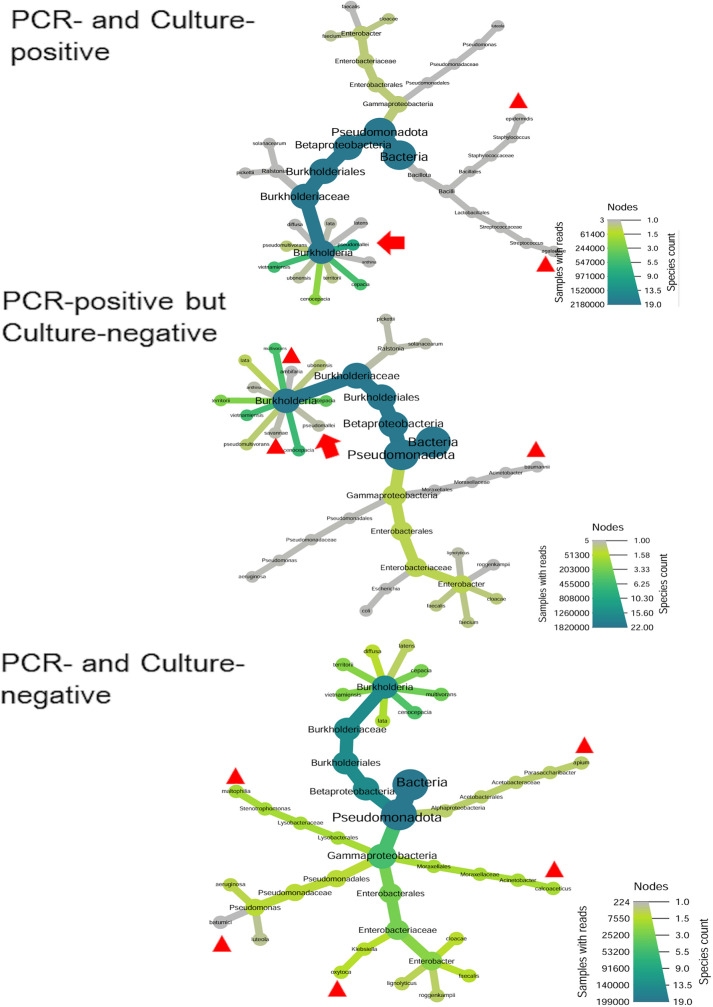
Bacterial Community Structures After 36 h of Incubation in Ashdown’s Broth. Soil supernatants prepared with sterile water and incubated overnight at 42°C were further cultured in Ashdown’s selective broth for 36 h. Total DNA was extracted and analyzed using full-length 16S rDNA sequencing. A Heat Tree visualization, generated using an R-based program from a combination of 6 individual datasets, depicts the bacterial community composition across 3 soil groups: (1) PCR and culture-positive, (2) PCR-positive but culture-negative*,* and (3) PCR- and culture-negative for *B. pseudomallei*. Red arrows indicate the position of *B. pseudomallei*, while triangles denote species uniquely present in a specific group. Node color intensity reflects the relative abundance (read counts), and node size corresponds to the number of taxa.

**Fig 6:**
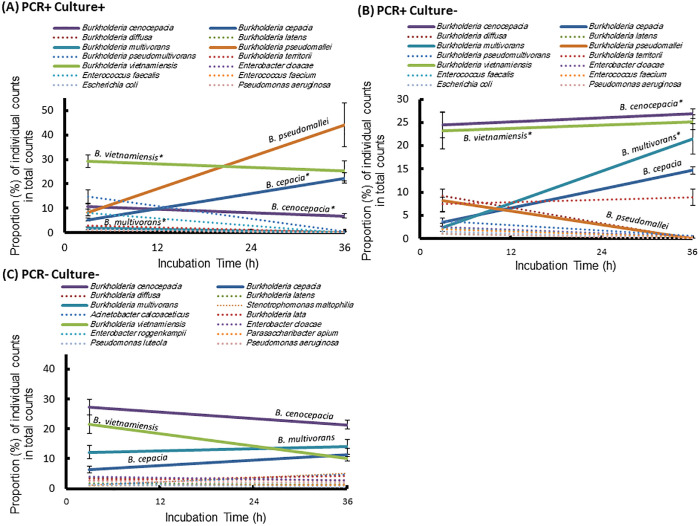
Dynamic Changes in Bacterial Populations During Incubation. Soil supernatants prepared with sterile water and incubated overnight at 42°C were further cultured in Ashdown’s selective broth for 2 and 36 h. The proportional changes (%) of individual read counts (n = 6 per group × 2 time points; Standard deviation > 0.05 is shown) relative to the total read counts are shown for the top 14 populations (each comprising >1% of the total community) across the following groups: PCR- and culture-positive (PCR+ Culture+) (A), PCR-positive but culture-negative (PCR+ Culture-) (B), and PCR- and culture-negative (PCR- Culture-) for *B. pseudomallei* (C). Asterisks (*) indicate bacterial taxa inversely correlated with *B. pseudomallei* abundance (Spearman’s ρ = -0.58 to -0.87, *p* < 0.05).

After a 2-h incubation by Ashdown’s selective broth, sequencing identified 55–75 distinct reads per group, including 20–23 species of the *Burkholderiaceae* family. *B. pseudomallei*-specific reads were detected in PCR-positive groups but were absent in PCR-negative samples (Fig 4). By 36 h of incubation, only 19–22 bacterial species were detected in each group, with 2–5 species uniquely present at low abundance (<1.2% of total reads). A total of 8–12 *Burkholderia* species were detected post-enrichment, including *B. pseudomallei* in the culture-enriched PCR-positive group (Fig 5). Among PCR-positive groups, the top 14 strains (each >1% abundance) were commonly shared. Notably, *B. pseudomallei* abundance increased to over 44% in the PCR- and culture-positive soil group (Fig 6A), but decreased to less than 0.04% in the PCR-positive but culture-negative soil group by 36 h (Fig 6B). The patterns of increasing and decreasing *B. pseudomallei* were inversely related to those of *B. multivorans*, *B. cenocepacia*, and *B. vietnamiensis* during incubation in both PCR-positive groups (Spearman’sρ = -0.58 to -0.87, *p* < 0.05). In the control groups (PCR- and culture-negative), *B. multivorans* still increased. Five of the top 14 species—*Stenotrophomonas maltophilia*, *Acinetobacter calcoaceticus*, *B. lata*, *Enterobacter roggenkampii*, *Parasaccharibacter apium*, *Pseudomonas luteola* (<5.2%, during incubation)—were present in controls but absent in both PCR-positive groups (Fig 6C).

### Bacterial Interaction *in vitro*

**Fig 7:**
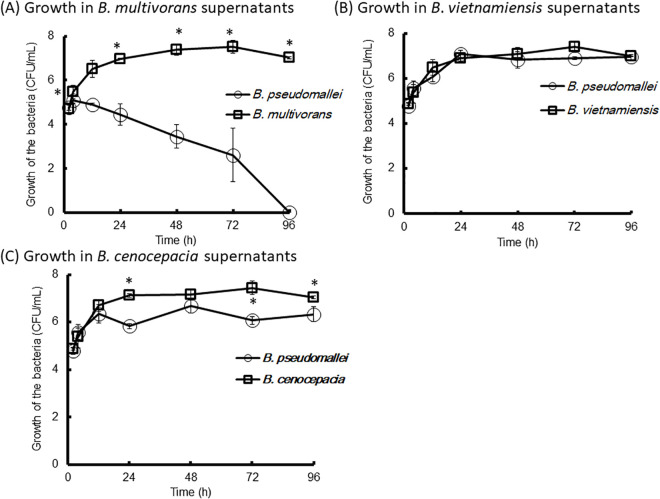
In vitro Inhibitory Effects of *Burkholderia* Supernatants on *B. pseudomallei.* The growth of *B. pseudomallei* VGH27 in filtered supernatants from *B. multivorans* NKNU07 **(A)**, *B. vietnamiensis* NKNU04 **(B)**, and *B. cenocepacia* NKNU05 **(C)**, is shown over the indicated time points. As controls, the bacterium was cultivated in its own filtered supernatant. Data represent 6 replicates per group (n = 6); asterisks (*) indicate statistically significant differences (P < 0.05).

To investigate potential antagonistic interactions within the *Burkholderia* genus, cell-free culture supernatants were prepared from three species, *B. multivorans* NKNU07, *B. vietnamiensis* NKNU04, and *B. cenocepacia* NKNU05 to assess inhibition of *B. pseudomallei* VGH27 growth. After 12 h, a decline in *B. pseudomallei* VGH27 was observed, and by 96 h, no viable *B. pseudomallei* VGH27 were detected in cultures grown in the cell-free supernatant of *B. multivorans* NKNU07. As controls, *B. multivorans* NKNU07 supernatants supported its own growth over the 96-h period (Fig 7A). No antagonistic interaction was observed between *B. vietnamiensis* NKNU04 and *B. pseudomallei* VGH27 (Fig 7B), whereas *B. cenocepacia* NKNU05 exhibited slight suppression of *B. pseudomallei* VGH27 (Fig 7C).

## Discussion

In this study, we investigated the geographical distribution of *B. pseudomallei*, the causative agent of melioidosis, in soil samples collected across Taiwan. Melioidosis, a disease currently seeking classification as a neglected tropical disease by the World Health Organization (WHO), poses a significant threat to populations in low-income regions with limited medical resources [2]. The geographical distribution of *B. pseudomallei* in endemic areas is defined by regions with reported non–travel–associated melioidosis cases and culture-confirmed or PCR-positive detections in the environment [13]. In southern Taiwan, melioidosis hotspots have been identified based on a high number of reported melioidosis cases (12.14 cases per 100,000 people) and PCR- or culture-confirmed *B. pseudomallei* from soil or water samples [7,21]. In this study, we found that PCR positivity was 15.1% in southern Taiwan, significantly higher than in other areas. Although eastern Taiwan has reported only 0.95 cumulative cases per 100,000 people from 2003 to 2024, it has relatively limited medical resources. Notably, 16 out of 163 soil samples (9.82%) tested positive for *B. pseudomallei* in this region. These findings suggest that nearly 500,000 residents in the region may face underrecognized risks of exposure to the pathogen.

The geographical distribution of *B. pseudomallei* is notably uneven at the regional scale. However, within areas where it is detected, PCR-positive locations are consistently found in clusters ranging from 3.3 to 48.9 km². Cluster 6 is situated along the Er-Ren River in Tainan City, and Cluster 7 is near Kaohsiung City in southern Taiwan; both locations have reported melioidosis outbreaks [11,21]. Local acquisition of melioidosis has been reported from the environment, typically from soil or freshwater sources-even in regions with few cases, such as Mississippi, USA [25]. Moreover, *B. pseudomallei* can be washed out with eroded soil during rainfall and conveyed to remote areas [26]. In this study, a vertical distribution survey revealed high PCR positivity in the southern land surface (Cluster 7), increasing the risks of human or animal exposure to the pathogen. Specific climatic conditions, such as heavy rainfall or typhoons, can generate aerosols from the land surface, further facilitating airborne transmission [24,27]. According to climatic changes, the positivity rate of *B. pseudomallei*-specific DNA on the land surface increased on rainy days and decreased during subsequent sunny days. Bacteria with the same molecular STs were identified across three soil layers (60 cm, 30 cm, and land surface) during a rainy event. This finding suggests that rainfall facilitates the dissemination of *B. pseudomallei* from deeper layers to the land surface, but the bacteria do not persist on the land surface for extended periods under sunny conditions.

In crop fields, factors such as nutrient levels, soil types, iron content, and fertilization have been reported to partially influence the growth and survival of *B. pseudomallei* [16,21,28–31]. Variations in plantation practices across extensive field studies from high to low altitudes limit the ability to establish clear links among the presence of *B. pseudomallei* DNA, agricultural practices, and fertilization methods in this study. Although standardized protocols were used, the required clearing of vegetation and slight leveling may have disrupted the rhizosphere, as this microenvironment is sensitive to sampling disturbance and may have influenced our observations [32].

Notably, over 95% of positive sites were located below a latitude of 24.3°N, extending beyond the typical 20°N to 20°S range where melioidosis is most prevalent, in alignment with predicted distributions [13]. Although distinct regional genetic clusters were identified in this study, the genetic type ST99, identical to human isolates, was widely found from northern to southern regions, indicating that *B. pseudomallei* exists in the environment across Taiwan [33]. Human exposure to environmental *B. pseudomallei* depends on the likelihood of the pathogen appearing on the land surface under certain climatic conditions or at a depth of 30 cm due to agricultural activities such as plowing [5]. Several studies published between 2007 and 2015 reported the isolation of *B. pseudomallei* from southern Taiwan, suggesting that the bacterium has persisted in harsh soil environments in the region for many years [7,11,24].

A selective Ashdown’s medium containing antibiotics was developed to isolate *B. pseudomallei* from clinical specimens and was later applied to environmental isolation [17,34]. *B. thailandensis* is commonly found in endemic areas of Thailand, Sierra Leone, Nigeria, Malaysia, Myanmar, and Papua New Guinea [35–38]. *B. humptydooensis*, which is genetically close to *B. thailandensis*, has been isolated from the Northern Territory of Australia [39–40]. However, neither *B. thailandensis* nor *B. humptydooensis* DNA was detected in our environmental samples.

The rhizosphere-associated bacteria *B. vietnamiensis*, *B. multivorans*, *B. cenocepacia*, and *B. pseudomallei* are known to inhabit agricultural soils, standing water, sludge, waste, and polluted environments [41–45]. In our cultivation-based experiments, these four bacterial species became predominant, likely due to selective pressures-such as antibiotics or crystal violet-that suppressed the growth of other bacterial populations [34]. An inverse distribution pattern was observed between *B. pseudomallei* and gentamicin-resistant *B. multivorans*, *B. cenocepacia,* and *B. vietnamiensis* during incubation in PCR-positive Culture-neative groups. For these cultures, while *B. multivorans*, *B. cenocepacia*, and *B. vietnamiensis* maintained high population levels, *B. pseudomallei* declined to <0.04%, suggesting possible bacterial competition. Consistent with previous findings [46], the growth of *B. pseudomallei* was inhibited by the supernatants of *B. multivorans* in our in vitro assays. *B. cenocepacia* also showed mild inhibitory effects*,* whereas *B. vietnamiensis* had no significant impact.

However, the culture-based detection method used in this study—relying on Ashdown’s medium—selectively enriches for gentamicin-resistant bacteria and may not accurately reflect the true composition or relative abundance of *Burkholderia* species in soil. In vitro competition assays were performed under controlled laboratory conditions, which may not replicate the complex and variable interactions present in natural soil environments, such as nutrient gradients, microhabitat structures, and broader microbial community dynamics. Additionally, *B. pseudomallei* in soil may be predated upon fungi, amoebae, nematodes, and slime molds [47–50] and its growth may be suppressed by bacterial competition through mechanisms such as secretion of antimicrobial compounds, phage-mediated lysis, toxin–antitoxin systems, or Type VI secretion system (T6SS)-mediated killing [50–52].

The observed inhibitory effect on *B. pseudomallei* in the presence of filtered *B. multivorans* supernatants is likely due to secreted factors from *B. multivorans*, such as secondary metabolites, broad-host-range bacteriophages, or toxic molecules that impair *B. pseudomallei* growth [53–54]. These findings suggest that *B. multivorans* can exert an antagonistic effect on *B. pseudomallei* through the production of inhibitory factors, particularly under favorable environmental conditions. However, the exact mechanism underlying this inhibition remains to be determined. Further analyses, such as mass spectrometry (MS) or transmission electron microscopy (TEM) of the supernatants, are needed to identify the active components involved. Interspecies interactions among *Burkholderia* species warrant further investigation to better understand competitive dynamics within this genus in soil environments.

Beyond microbial factors, dynamic bacterial community structures can emerge under agricultural practices such as plowing and irrigation, as well as climatic factors like UV radiation, temperature, wind, and rainfall [55]. Climate change, in particular, has been shown to alter soil microbial communities and increase the risk of soil-borne diseases [56]. The occurrence and dissemination of melioidosis depend on the presence of environmental *B. pseudomallei*, particularly when it emerges in high abundance at the soil surface [24]. This disease is gradually spreading from endemic to non-endemic areas, often facilitated by flooding due to heavy rains [57]. Notably, melioidosis frequently reemerges in multiple regions following severe climate events and natural disasters [56,58]. Our finding that *B. pseudomallei* appears on the land surface during rainy periods offers a potential explanation for the observation that melioidosis outbreaks in Taiwan have typically followed heavy rainfall associated with typhoons [21].

In conclusion, our findings reveal that *B. pseudomallei* is distributed across Taiwan, including in regions with few reported cases, and that its surface emergence is strongly influenced by rainfall. The presence of competing soil bacteria, such as *B. multivorans*, may contribute to the underestimation of *B. pseudomallei* in culture-based environmental surveys, particularly in southern Taiwan.

## Supporting information

S1 Fig Coordinates of Sampling sites on southern land During a rainy day. Sampling was conducted on the southern land surface on a rainy day. The coordinates of the sampling sites used for vertical distribution and bacterial community analysis are shown. Each square represents a 0.64 m² sampling unit. Gray squares indicate PCR-negative soils, while yellow squares indicate PCR-positive soils that did not yield viable *B. pseudomallei.* Each sequence type (ST) type is represented by different colored squares at the bottom (see Results for details). The yellow square with a solid black outline denotes a PCR-positive soil sample that did not yield viable *B. pseudomallei* across all three sampled layers (60 cm, 30 cm, and the surface layer).
(TIF)

S2 Fig Proportional Changes in PCR-Positive Samples and Sequence Types (STs) in the Northern Region. The proportions (%) of PCR-positive samples (PCR + , green), PCR-positive but culture-negative samples within a single soil layer (PCR+ Culture − , yellow), and PCR-positive but culture-negative samples across all three soil layers (PCR+ Culture − / − / − , yellow with a solid black outline) are shown for the initial sunny day (Sunny 0), the rainy day (Rainy), and the first (Sunny 1) and second sunny (Sunny 2) days following rainfall. The proportional distribution of STs isolated using a fixed-interval vertical sampling strategy in the northern region throughout the rainfall event is also shown below each bar chart for each soil depth. Percentages do not sum to exactly 100% because multiple STs were occasionally isolated from the same sample, and some samples were PCR-positive but culture-negative.
(TIF)

S3 Fig Proportional Changes in PCR-Positive Samples and Sequence Types (STs) in the Central Region. The proportions (%) of PCR-positive samples (PCR + , green), PCR-positive but culture-negative samples within a single soil layer (PCR+ Culture − , yellow), and PCR-positive but culture-negative samples across all three soil layers (PCR+ Culture − / − / − , yellow with a solid black outline) are shown for the initial sunny day (Sunny 0), the rainy day (Rainy), and the first (Sunny 1) and second sunny (Sunny 2) days following rainfall. The proportional distribution of STs isolated using a fixed-interval vertical sampling strategy in the central region throughout the rainfall event is also shown below each bar chart for each soil depth. Percentages do not sum to exactly 100% because multiple STs were occasionally isolated from the same sample, and some samples were PCR-positive but culture-negative.
(TIF)

S4 Fig Proportional Changes in PCR-Positive Samples and Sequence Types (STs) in the Southern Region. The proportions (%) of PCR-positive samples (PCR + , green), PCR-positive but culture-negative samples within a single soil layer (PCR+ Culture − , yellow), and PCR-positive but culture-negative samples across all three soil layers (PCR+ Culture − / − / − , yellow with a solid black outline) are shown for the initial sunny day (Sunny 0), the rainy day (Rainy), and the first (Sunny 1) and second sunny (Sunny 2) days following rainfall. The proportional distribution of STs isolated using a fixed-interval vertical sampling strategy in the southern region throughout the rainfall event is also shown below each bar chart for each soil depth. Percentages do not sum to exactly 100% because multiple STs were occasionally isolated from the same sample, and some samples were PCR-positive but culture-negative.
(TIF)

S5 Fig Proportional Changes in PCR-Positive Samples and Sequence Types (STs) in the Eastern Region. The proportions (%) of PCR-positive samples (PCR + , green), PCR-positive but culture-negative samples within a single soil layer (PCR+ Culture − , yellow), and PCR-positive but culture-negative samples across all three soil layers (PCR+ Culture − / − / − , yellow with a solid black outline) are shown for the initial sunny day (Sunny 0), the rainy day (Rainy), and the first (Sunny 1) and second sunny (Sunny 2) days following rainfall. The proportional distribution of STs isolated using a fixed-interval vertical sampling strategy in the eastern region throughout the rainfall event is also shown below each bar chart for each soil depth. Percentages do not sum to exactly 100% because multiple STs were occasionally isolated from the same sample, and some samples were PCR-positive but culture-negative.
(TIF)

S1 Table PCR and culture detection for imported soil samples in pre-test. (DOCX)

S2 Table Sample sizes, sampling region, PCR positive rates and specific DNA concentration. (DOCX)

S3 Table Cumulative melioidosis incidence from 2003 to 2024 across Taiwan. (DOCX)
