# Supplementary figures and images for: Geographical distribution of Burkholderia pseudomallei in Taiwanese croplands and the influence of bacterial community interactions on its incubation viability
Source: PLoS Negl Trop Dis. 2025 Oct 22;19(10):e0013640. doi: 10.1371/journal.pntd.0013640 (PMC12574894; doi:10.1371/journal.pntd.0013640)

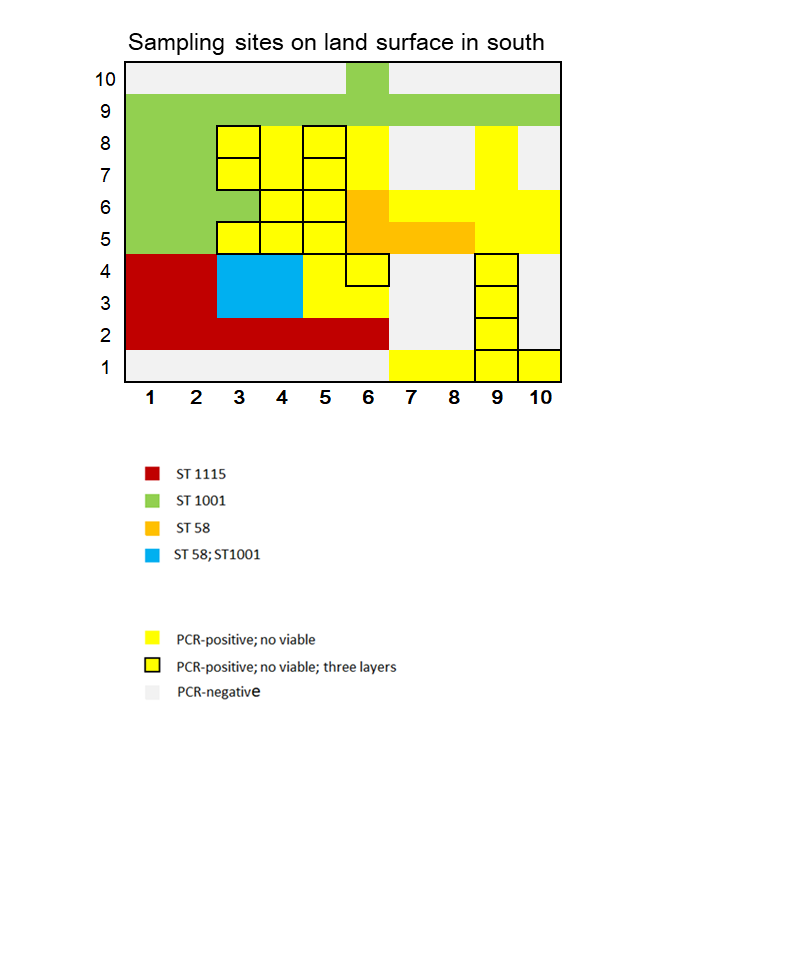

Supplement: S1 Fig — Sampling was conducted on the southern land surface on a rainy day. The coordinates of the sampling sites used for vertical distribution and bacterial community analysis are shown. Each square represents a 0.64 m² sampling unit. Gray squares indicate PCR-negative soils, while yellow squares indicate PCR-positive soils that did not yield viable B. pseudomallei. Each sequence type (ST) type is represented by different colored squares at the bottom (see Results for details). The yellow square with a solid black outline denotes a PCR-positive soil sample that did not yield viable B. pseudomallei across all three sampled layers (60 cm, 30 cm, and the surface layer). (TIF) [file pntd.0013640.s001.tif]

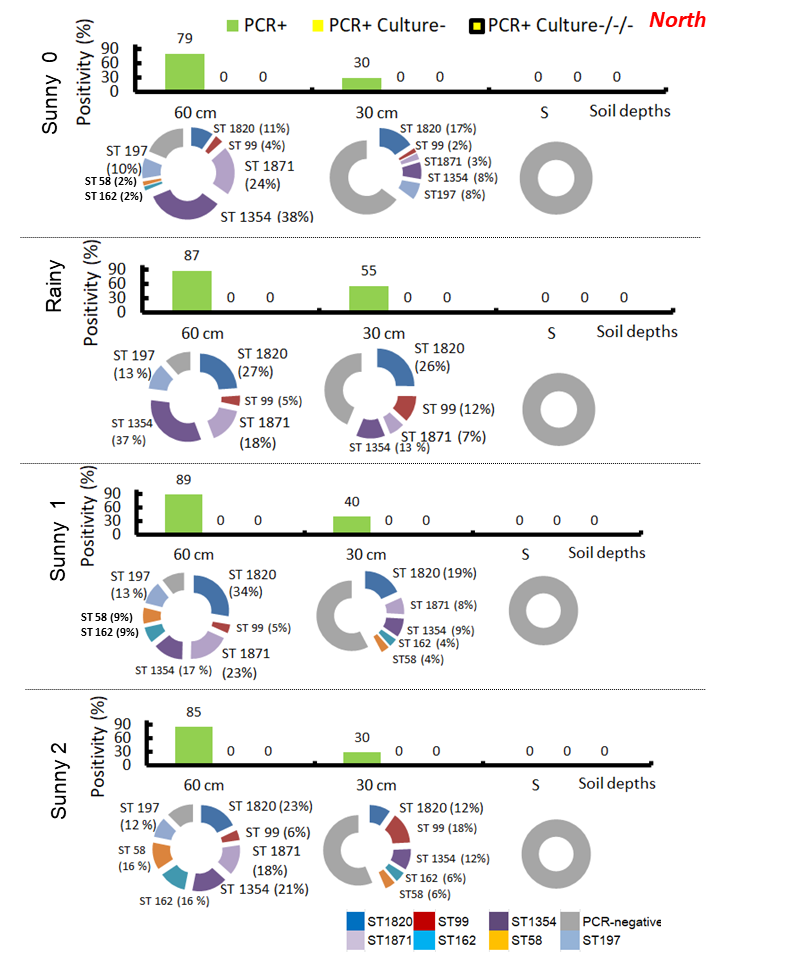

Supplement: S2 Fig — The proportions (%) of PCR-positive samples (PCR + , green), PCR-positive but culture-negative samples within a single soil layer (PCR+ Culture − , yellow), and PCR-positive but culture-negative samples across all three soil layers (PCR+ Culture − / − / − , yellow with a solid black outline) are shown for the initial sunny day (Sunny 0), the rainy day (Rainy), and the first (Sunny 1) and second sunny (Sunny 2) days following rainfall. The proportional distribution of STs isolated using a fixed-interval vertical sampling strategy in the northern region throughout the rainfall event is also shown below each bar chart for each soil depth. Percentages do not sum to exactly 100% because multiple STs were occasionally isolated from the same sample, and some samples were PCR-positive but culture-negative. (TIF) [file pntd.0013640.s002.tif]

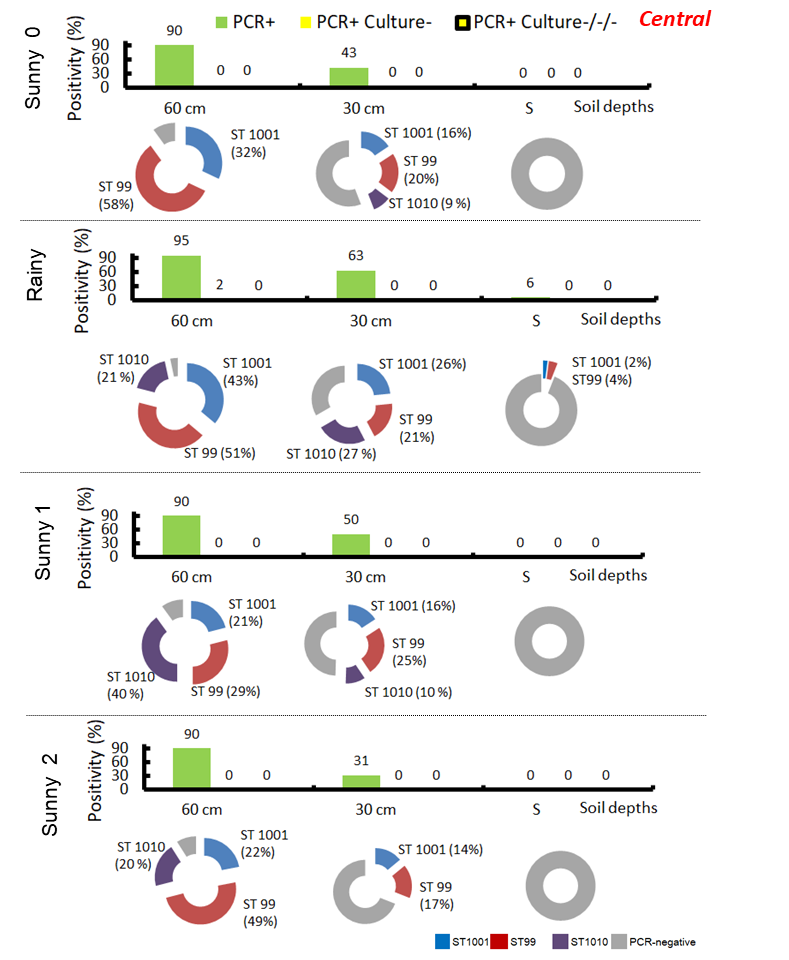

Supplement: S3 Fig — The proportions (%) of PCR-positive samples (PCR + , green), PCR-positive but culture-negative samples within a single soil layer (PCR+ Culture − , yellow), and PCR-positive but culture-negative samples across all three soil layers (PCR+ Culture − / − / − , yellow with a solid black outline) are shown for the initial sunny day (Sunny 0), the rainy day (Rainy), and the first (Sunny 1) and second sunny (Sunny 2) days following rainfall. The proportional distribution of STs isolated using a fixed-interval vertical sampling strategy in the central region throughout the rainfall event is also shown below each bar chart for each soil depth. Percentages do not sum to exactly 100% because multiple STs were occasionally isolated from the same sample, and some samples were PCR-positive but culture-negative. (TIF) [file pntd.0013640.s003.tif]

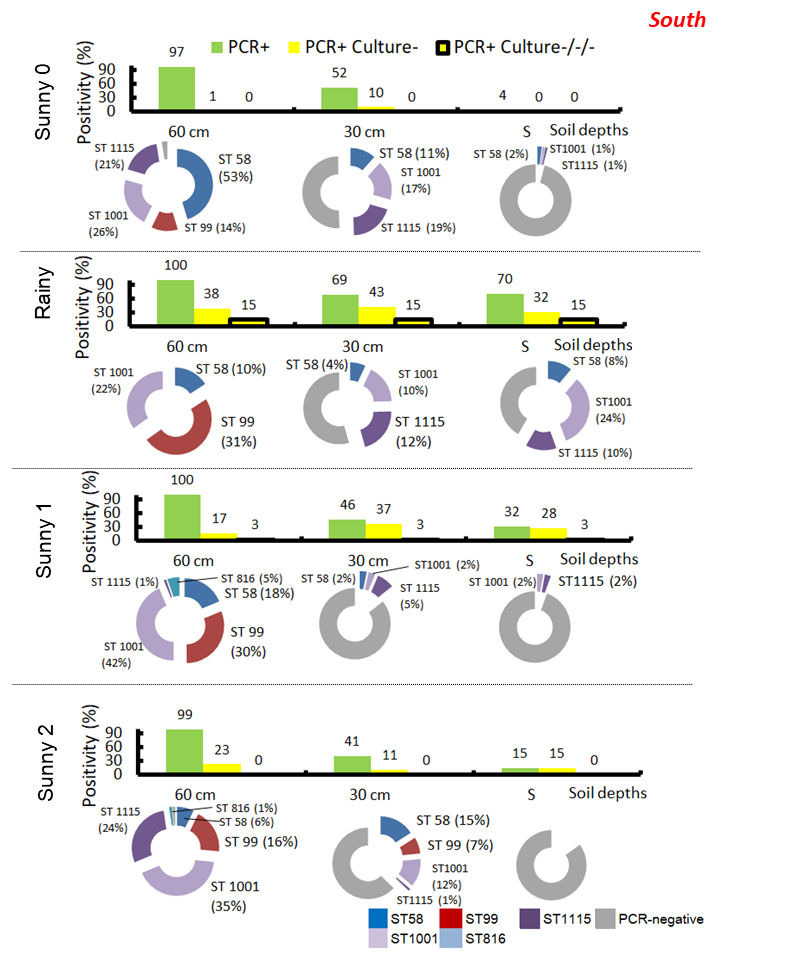

Supplement: S4 Fig — The proportions (%) of PCR-positive samples (PCR + , green), PCR-positive but culture-negative samples within a single soil layer (PCR+ Culture − , yellow), and PCR-positive but culture-negative samples across all three soil layers (PCR+ Culture − / − / − , yellow with a solid black outline) are shown for the initial sunny day (Sunny 0), the rainy day (Rainy), and the first (Sunny 1) and second sunny (Sunny 2) days following rainfall. The proportional distribution of STs isolated using a fixed-interval vertical sampling strategy in the southern region throughout the rainfall event is also shown below each bar chart for each soil depth. Percentages do not sum to exactly 100% because multiple STs were occasionally isolated from the same sample, and some samples were PCR-positive but culture-negative. (TIF) [file pntd.0013640.s004.tif]

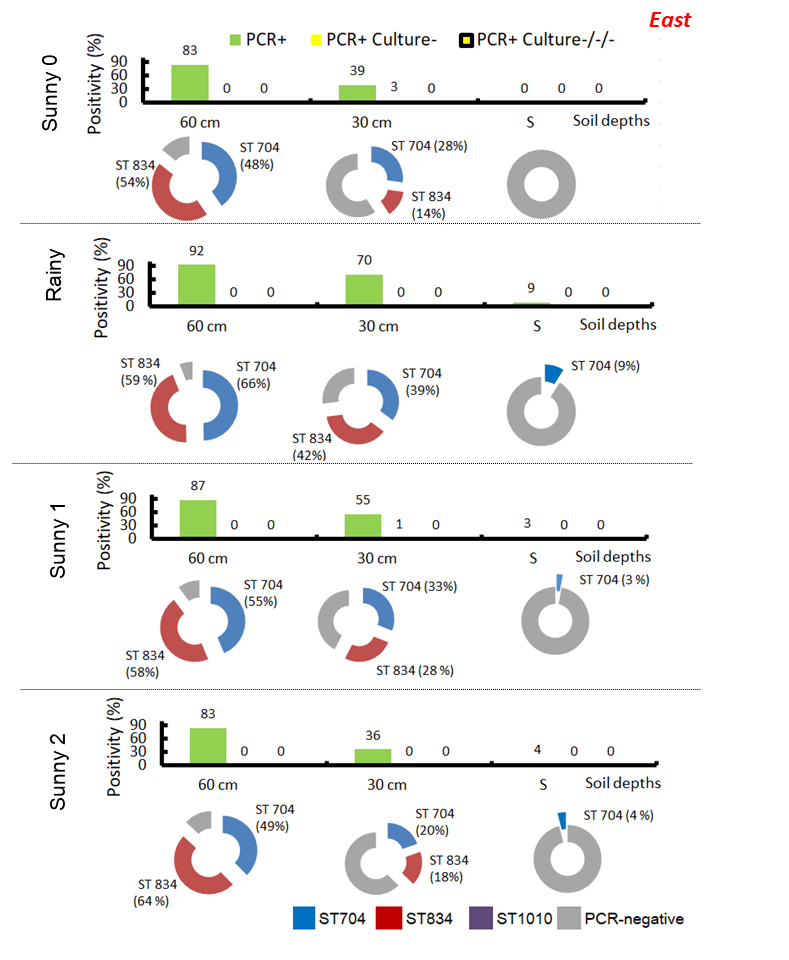

Supplement: S5 Fig — The proportions (%) of PCR-positive samples (PCR + , green), PCR-positive but culture-negative samples within a single soil layer (PCR+ Culture − , yellow), and PCR-positive but culture-negative samples across all three soil layers (PCR+ Culture − / − / − , yellow with a solid black outline) are shown for the initial sunny day (Sunny 0), the rainy day (Rainy), and the first (Sunny 1) and second sunny (Sunny 2) days following rainfall. The proportional distribution of STs isolated using a fixed-interval vertical sampling strategy in the eastern region throughout the rainfall event is also shown below each bar chart for each soil depth. Percentages do not sum to exactly 100% because multiple STs were occasionally isolated from the same sample, and some samples were PCR-positive but culture-negative. (TIF) [file pntd.0013640.s005.tif]
