# Supplementary material for: Geographical distribution of Burkholderia pseudomallei in Taiwanese croplands and the influence of bacterial community interactions on its incubation viability
Source: PLoS Negl Trop Dis. 2025 Oct 22;19(10):e0013640. doi: 10.1371/journal.pntd.0013640 (PMC12574894; doi:10.1371/journal.pntd.0013640)
Supplement: S1 Table — (DOCX) [file pntd.0013640.s006.docx]

| S1 Table. PCR and culture detection for imported soil samples in pre-test. | | |
| --- | --- | --- |
| Proportion (%) of PCR- and Culture-positive for *B. pseudomallei* | | |
|  | Soil sources | |
|  | Darwin | Ubon Ratchathani |
| Sample numbers | 30 | 30 |
| PCR-positivity (%) | 96.6 (29/30) | 100 (30/30) |
| Culture-confirmed *B. pseudomallei* in soils (%)*^a^* | 96.6 (29/30)^b^ | 100 (30/30) |
| ^a,^ Methodology for culture and PCR detection is described in the Methodology section. | | |
| ^b,^ One imported sample from Darwin was negative by both PCR and culture. | | |
