# Supplementary material for: Geographical distribution of Burkholderia pseudomallei in Taiwanese croplands and the influence of bacterial community interactions on its incubation viability
Source: PLoS Negl Trop Dis. 2025 Oct 22;19(10):e0013640. doi: 10.1371/journal.pntd.0013640 (PMC12574894; doi:10.1371/journal.pntd.0013640)
Supplement: S2 Table — (DOCX) [file pntd.0013640.s007.docx]

**S2 Table. Sample sizes, sampling region, PCR positive rates and specific DNA concentration**

|  | Sampling Region | | | | | | | | | |
| --- | --- | --- | --- | --- | --- | --- | --- | --- | --- | --- |
|  | Northern | Central | | | | Southern | | Eastern | | Northeastern |
| Sampling number | 240 | 368 | | | | 212 | | 163 | | 28 |
| plantation (PCR positive) |  |  |  |  |  |  |  |  |  |  |
| Fallow | 27 (0) | 23 (0) | | | | 23 (3) | | 6 (0) | | 5 |
| Fruit tree | 20 (0) | 41 (2) | | | | 80 (2) | | 88 (6) | | 1 |
| Paddy | 152 (5) | 159 (15) | | | | 62 (18) | | 53 (8) | | 15 |
| Herbaceous plant | 41 (0) | 145 (13) | | | | 47 (9) | | 16 (2) | | 7 |
| PCR-positive rate (%) | 2.1% (5/240) | 8.2% (30/368) | | | | 15.1% (32/212) | | 9.8% (16/163) | | 0% |
| Total numbers of clusters | N=1 | N=4 | | | | N=2 | | N=2 | |  |
| Designed number of the cluster | 1^#^ | 2 | 3 | 4 | 5^#^ | 6 | 7^#^ | 8^#^ | 9 |  |
| Area size (Km^2^) | 23.81 | 28.24 | 9.25 | 48.89 | 74 | 12.81 | 16.16 | 3.3 | 11.12 |  |
| Numbers of scattered points | 0 | 1 | | | | 2 | | 2 | |  |
| Average specific DNA concentration | 1528±217 | 1413±131 | | | | 1450±252 | | 1490±155 | | ND* |
| (copies/g) |  |  |  |  |  |  |  |  |  |  |

*, Not detected

^#^, Selected for vertical distribution analysis
