# Supplementary material for: Geographical distribution of Burkholderia pseudomallei in Taiwanese croplands and the influence of bacterial community interactions on its incubation viability
Source: PLoS Negl Trop Dis. 2025 Oct 22;19(10):e0013640. doi: 10.1371/journal.pntd.0013640 (PMC12574894; doi:10.1371/journal.pntd.0013640)
Supplement: S3 Table — (DOCX) [file pntd.0013640.s008.docx]

**S3 Table.** **Cumulative melioidosis incidence from 2003 to 2024 across Taiwan.**

| Region^#^ | Cumulative cases | Average population | Incidence (per 100,000) | 95% CI* Lower | 95% CI Upper |
| --- | --- | --- | --- | --- | --- |
| North | 35 | 10,822,696 | 0.32 | 0.22 | 0.43 |
| Central | 95 | 5,957,964 | 1.59 | 1.28 | 1.93 |
| South | 653 | 5,379,302 | 12.14 | 11.21 | 13.09 |
| East | 5 | 525,592 | 0.95 | 0.19 | 1.90 |
| Northeast | 2 | 449,212 | 0.44 | 0.0 | 1.11 |
| Total (Taiwan) | 790 | 23,134,766 | 3.42 | 3.18 | 3.65 |

# Districts and/or cities: Taipei, Keelung, New Taipei, Hsinchu, Taoyuan, and Miaoli in Northern; Changhua, Nantou, Taichung, Chiayi, and Yunlin in Central; Tainan, Kaohsiung, and Pingtung in Southern; Hualien and Taitung in Eastern; and Yilan in Northeastern region across Taiwan.

* 95% CI: 95% confidence interval for incidence per 100,000 people.
